# Near-infrared-dye labeled tumor vascular-targeted dimer GEBP11 peptide for image-guided surgery in gastric cancer

**DOI:** 10.3389/fonc.2022.885036

**Published:** 2022-11-24

**Authors:** Zuhong Tian, Shuhui Liang, Xinmin Zhou, Hui Luo, Miaomiao Tian, Xianghan Zhang, Changcun Guo, Jing Zhang

**Affiliations:** ^1^ State Key Laboratory of Cancer Biology & XiJing Hospital of Digestive Diseases, Air Force Medical University, Xi’an, China; ^2^ Engineering Research Center of Molecular-imaging and Neuroimaging of Ministry of Education, School of Life Science and Technology, Xidian University, Xi’an, China

**Keywords:** fluorescence imaging, image-guided surgery, gastric cancer, GEBP11 peptide, near-infrared

## Abstract

**Introduction:**

Positive resection margins occur in about 2.8%-8.2% gastric cancer surgeries and is associated with poor prognosis. Intraoperative guidance using Nearinfrared (NIR) fluorescence imaging is a promising technique for tumor detection and margin assessment. The goal of this study was to develop a tumor-specific probe for real-time intraoperative NIR fluorescence imaging guidance.

**Methods:**

The tumor vascular homing peptide specific for gastric cancer, GEBP11, was conjugated with a near-infrared fluorophore, Cy5.5. The binding specificity of the GEBP11 probes to tumor vascular endothelial cells were confirmed by immunofluorescent staining. The ability of the probe to detect tumor lesions was evaluated in two xenograft models. An orthotopic gastric cancer xenograft model was used to evaluate the efficacy of the GEBP11 NIR probes in real-time surgical guidance.

**Results:**

In vitro assay suggested that both mono and dimeric GEBP11 NIR probes could bind specifically to tumor vascular epithelial cells, with dimeric peptides showed better affinity. In tumor xenograft mice, live imaging suggested that comparing with free Cy5.5 probe, significantly stronger NIR signals could be detected at the tumor site at 24-48h after injection of mono or dimeric GEBP11 probes. Dimeric GEBP11 probe showed prolonged and stronger NIR signals than mono GEBP11 probe. Biodistribution assay suggested that GEBP11 NIR probes were enriched in gastric cancer xenografts. Using dimeric GEBP11 NIR probes in real-time surgery, the tumor margins and peritoneal metastases could be clearly visualized. Histological examination confirmed the complete resection of the tumor.

**Conclusion:**

(GEBP11)2-ACP-Cy5.5 could be a potential useful probe for intraoperative florescence guidance in gastric cancer surgery.

## Introduction

Gastric cancer is the third leading cause of cancer-related deaths worldwide with over 1 million estimated new cases detected annually (1). Curative surgery constitutes the mainstay for gastric cancers, even though targeted therapies and immunotherapy are widely used (2–4). Surgery for gastric cancer aims at R0 resection with negative margins and adequate lymphadenectomy. Following guidelines on margin length, R0 resection could be achieved in the majority of gastric cancer patients. However, positive resection margin is reported in about 2.8%-8.2% gastric cancer patients who underwent gastrectomy and is associated with poor clinical outcomes (5, 6). Intraoperative fluorescence or endoscopic guidance have been used to assist the gastrectomy procedures (7, 8). However, surgeons still primarily rely on white light to determine the margins in gastric cancer surgery. Powerful intraoperative image guidance will be helpful for fast and accurate visualization of tumors during surgery and minimize R1 resection.

Near-infrared (NIR) fluorescence imaging holds great promise for image-guided surgery due to relatively low autofluorescence and deep photon penetration as well as high sensitivity without risk of radiation exposure (9). With the assistance of NIR fluorescence imaging, surgical field can be color coded and margins between the tumor and normal tissues can be well visualized (10). When combined with appropriate contrast agents, NIR fluorescence imaging can provide real-time image guidance to ensure successful removal of all the cancerous tissue and decrease complications. NIR fluorescence probes have been successful used in intraoperative tumor imaging and sentinel lymph node (SLN) mapping of various types of cancers such as ovary cancer, prostate cancer, colon and rectum cancer and gastric cancer (11–13). Currently, indocyanine green (ICG)-based fluorescence imaging has been used for real-time anatomy assessment and intraoperative lymphography (7, 14). Though promising, ICG is not cancer-specific, and could yield false positive or false negative results (15, 16). Human epidermal receptor 2 (HER2) and folate receptor have also been targeted for real-time intraoperative molecular imaging of gastric cancer (17). But their expression in the cancer tissue significantly affects the performance of these methods.

Using *in vivo* phage display, we have identified a peptide, GEBP11, which specifically binds to the tumor vasculature of human gastric cancer (18). In our previous studies, I131 or FITC labeled GEBP11 probes showed high affinity and specificity to the vasculature of gastric cancer, which could be used for *in vivo* imaging and targeted therapy (19, 20). In the present study, mono and dimeric GEBP11 peptides were labeled with NIR fluorophore dye, Cy5.5. These probes were verified *in vitro* and *in vivo* for their specificity and affinity. Then dimeric GEBP11 NIR probe was used for intraoperative NIR fluorescence imaging guidance in gastric cancer xenograft mouse models. Margins of the surgical specimens was examined to confirm complete resection.

## Materials and methods

### Synthesis and fluorescence labeling of (GEBP11)_2_-ACP and GEBP11

Both mono and dimeric GEBP11 peptide were biochemically synthesized by Qiangyao biotechnology company (Shanghai, China). Dimeric GEBP11 peptide was prepared by covalently crosslinking single peptides with an artificial peptide (ACP) linker, to form (GEBP11)_2_-ACP. For labeling, GEBP11 peptide (2.12 mg, 2 mmol) or (GEBP11)_2_-ACP (4.43 mg, 2 mmol) and TEA (12 μL, 0.08 mmol) were dissolved in 1ml DMSO, with a 2-fold molar excess of Cy5.5-NHS (2 mg, 4 mmol) separately. The mixture was further protected from light and rotated at room temperature overnight. After completion of reaction, the mixture was lyophilized using a freeze-dryer to remove DMSO. Next, the product was dissolved in 2 mL PBS in dialysis bag and dialyzed (1L PBS × 3) to remove the unconjugated Cy5.5. Finally, the solution was lyophilized to obtain Cy5.5 labeled probes. The probes were characterized by high resolution mass spectrometer to confirm their purities (HRMS, ESI, positive mode; Cy5.5-GEBP11: calculated *M_r_* =1999.7, found m/z =1997.6405 ([*M_r_*]). Cy5.5-(GEBP11)_2_: calculated *M_r_* = 3210.4, found m/z = 1605.3002 [(*M_r_+*H^+^)/2]).

### Cell lines

Human umbilical vein endothelial cells (HUVECs) and human gastric adenocarcinoma cell line SGC7901 used for *in vitro* targeting test were preserved in our laboratory. SGC7901 cells constitutively expressing luciferase or GFP (SGC7901-Luc or SGC7901-GFP) were established as previously reported (21), dual transfected cell lines were prepared for establishing tumor xenograft models. All mentioned cells were cultured in RPMI 1640 medium, supplemented with 10% fetal calf serum (Gibco, USA), 100 μg/mL streptomycin and 100 units/mL penicillin.

### *In vitro* specificity and affinity assay

Previous studies have demonstrated that co-culture of the cancer cells with vascular endothelial cells could create a similar microenvironment with the situation in the solid tumor mass (22, 23). SGC7901 cells and HUVECs were cocultured as previously described (18). Co- HUVECs (co-cultured HUVECs), HUVECs and SGC7901 cells were harvested at log-growth phase and characterized by bench top fluorescent microscopy. Cells were incubated with 1μM GEBP11-Cy5.5 or (GEBP11)_2_-ACP-Cy5.5 for 6h at 37°C in a humidified atmosphere with 5% CO_2_. Binding affinity of the probes to cultured cells were detected by laser scanning confocal microscope (FV3000, Olympus, Japan). Nucleus were re-stained with ProLong™ Gold Antifade Mountant with DAPI (Invitrogen, USA) at room temperature. For binding specificity assessment, cells were pretreated with 25μM unlabeled GEBP11 for 30min at 37°C before incubating with the labeled probes.

### Animal tumor xenograft models

All animal experiments were conducted in compliance with protocols approved by the Institute of Animal Use Committee of Air Force Medical University. All gastric cancer xenograft models were established in 6–8 week old female athymic nude mice. The SGC7901-Luc or SGC7901-GFP cell suspension at a concentration of 2 × 10^6^ cells/mL was prepared. For subcutaneous xenograft model, SGC7901-GFP cells suspension (100μL/mouse) were injected subcutaneously into the right upper limb (for *in vivo* binding affinity test) or lower flank of mouse (for *in vivo* biodistribution assay). The orthotopic gastric cancer xenograft models were established as described previously (21), In brief, open abdominal surgery was performed on the mice under general anesthesia, SGC7901-Luc cells suspension (100μL/mouse) were injected into the subserosa layer of the stomach.

Tumor growth was monitored by Caliper IVIS Lumina II (PerkinElmer, MA, USA). GFP fluorescence imaging was performed in subcutaneous xenograft model. Bioluminescence imaging (BLI) was performed to monitor tumor growth in the orthotopic xenograft models. Because of its higher penetrability and sensitivity, peritoneal metastasis can be visualized clearly as well. Before BLI was acquired, mice were injected with D-luciferin (150 mg/kg) by intraperitoneal injection and anesthetized with 2% isoflurane. Living Image software 4.7.3 (IVIS) was used to quantify bioluminescence signals.

All mice were fed irradiated alfalfa-free rodent diet (Envigo Teklad, Madison, WI) to reduce background in stomach and gut for one week prior to the imaging experiments. The mice models were used for *in vivo* imaging and surgical procedures when xenograft diameter reached 5 to 10 mm.

### Live imaging and biodistribution assays

Xenograft mice were injected with 200 ul GEBP11-Cy5.5, (GEBP11)_2_-ACP-Cy5.5 or free Cy5.5 (5 µM in PBS) *via* tail veins. Live NIR fluorescence imaging was performed at different time points. NIR Fluorescence intensity was quantified by region-of-interest (ROI) measurement using Living Image software 4.7.3 (IVIS).

Subcutaneous tumor xenograft models were used to detect the biodistribution of NIR signals. Mice were euthanized at 24h after injection of the labeled probes or free Cy5.5. All assays were done in triplicates. Then tumor xenografts and main organs (heart, lung, kidney, bone, muscle, brain, spleen, liver, skin and intestines) were harvested. Living Image version 4.7.3 Software (IVIS) was used to quantify the NIR fluorescence intensity at selected ROIs. All ROI data were collected after subtracting background autofluorescence. Differences in the fluorescence intensities of the ROIs between different probes were statistically analyzed.

### Real-time NIR fluorescence imaging-guided resection and histological analysis

Orthotopic gastric cancer xenograft mice were anesthetized with a ketamine/xylazine mixture *via* intraperitoneal injection 24h after injection of (GEBP11)_2_-ACP-Cy5.5 probe. Real-time NIR fluorescence imaging was used to guide the removal of the primary cancer lesion and potential metastatic lesions. After resection, all surgical specimens and surgical beds were examined by NIR fluorescence imaging to confirm complete resection. To compare the efficacy of the dimer GEBP11 NIR imaging, BLI imaging pictures were taken before and after resection, the findings of BLI imaging were kept blind to the researcher who performed the surgery.

Resected samples were embedded in optimum cutting temperature (OCT) compound (Leica, Germany), and 10μm-thick frozen tissue sections were cut on a cryostat microtome (Leica, Germany). The sections were fixed immediately with precooled isoacetone for 30min and stored at –20 °C. Hematoxylin and Eosin (H&E) staining was used for histological analysis. Tumor margins defined by H&E staining and NIR fluorescence were compared. All slides were scanned by using Pannoramic-250 Flash II slide-scanner (3D-Histech, Budapest, Hungary) and analyzed by Case viewer software (3D-Histech, Budapest, Hungary). Mouse tumor vascular endothelial cells were stained by FITC-labeled rat anti-mouse CD31 (89C2) antibody (CST, Boston, USA). Fluorescence microscopy was used to validate the colocalization of (GEBP11)_2_-ACP-Cy5.5 probe and tumor microvessel. Fluorescence microscopic images were acquired at 100x magnification by using Echo Revolve upside-down integrated fluorescence microscope (ECHO, USA).

### Statistical analysis

Quantitative data were expressed as means± standard error. Student t-test was used to compare mean values. Statistical analyses were performed using the Graphpad Prism 8.0.2(263). All the statistical analyses were two-tailed. *P*-value<0.05 were considered statistical significance (*p<0.05, **p<0.01, ***p<0.005, ****p<0.001.)

## Results

### Preparation of GEBP11 NIR probes and *in vitro* binding assays

The structures and mass spectroscopy analysis results were shown in **Figure S1**. The purities of synthesized peptides were 99.2% for GEBP11 and 95.8% for (GEBP11)_2_-ACP-Cy5.5. The labelling efficiency of Cy5.5-GEBP11 and (GEBP11)_2_-ACP-Cy5.5 were 90% and 75%, respectively.

**Figure 1:**
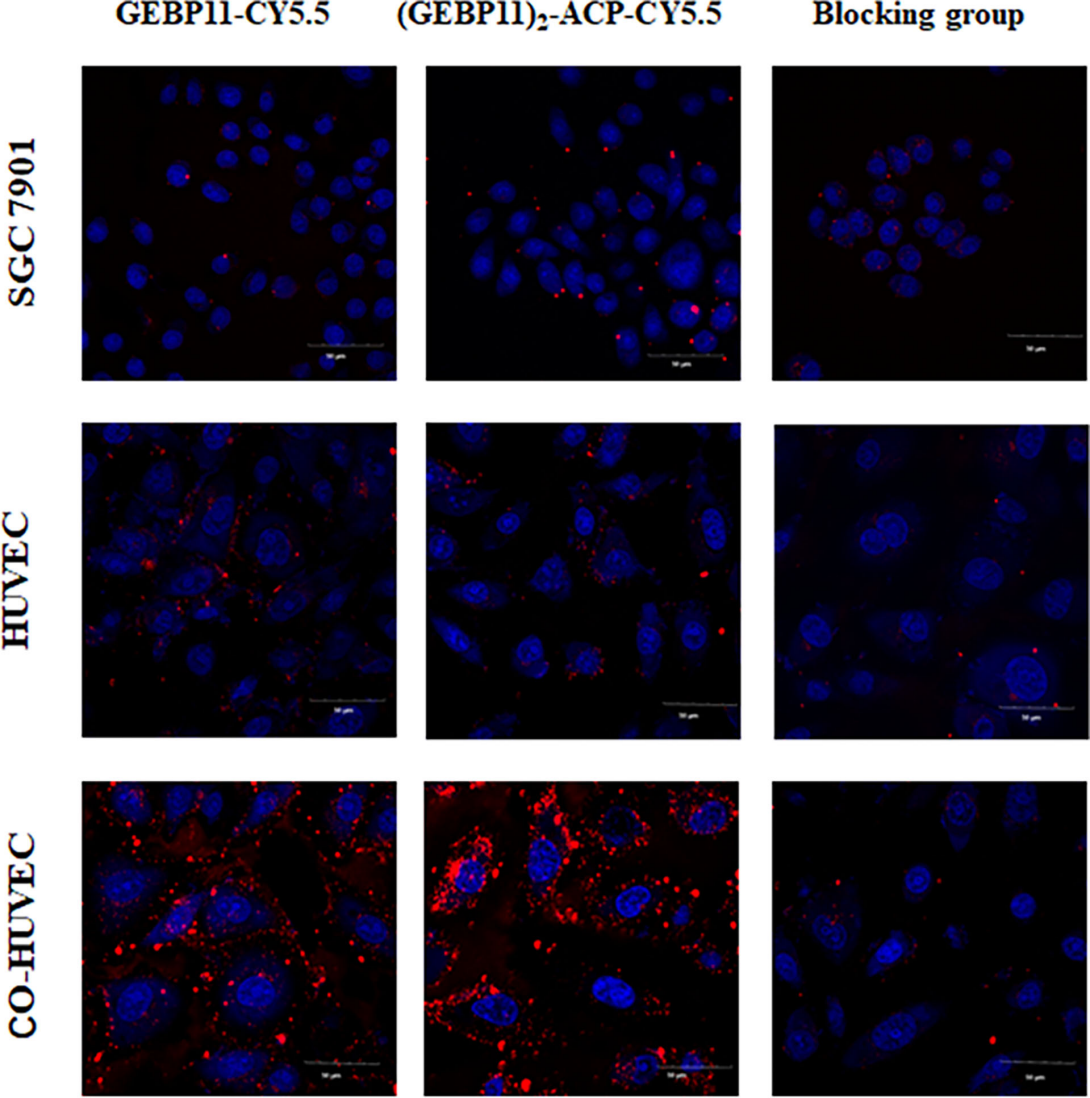
*In vitro* binding affinity of (GEBP11)_2_-ACP-Cy5.5 or GEBP11-Cy5.5. Confocal immunofluorescence of Cy5.5 labeled GEBP11 probes showed that a high level of (GEBP11)_2_-ACP-Cy5.5 accumulated in the Co-HUVECs. The competitive inhibition assay demonstrated the suppressive effect of GEBP11-Cy5.5 binding to Co-HUVECs by GEBP11. Cells were stained with DAPI in blue, and Cy5.5 labeled probes colored in red. Scale bar, 50μm.

After incubation with GEBP11-Cy5.5 or (GEBP11)2-ACP-Cy5.5 probes, Co-HUVECs showed strong fluorescence signal, while only scarce signal could be detected on HUVECs or SGC7901. (GEBP11)_2_-ACP-Cy5.5 showed much higher signal intensity than GEBP11-Cy5.5. Blocking with unlabeled GEBP11 significantly inhibited the binding of labeled probes to the Co-HUVECs (**Figure 1**). These findings suggested that both GEBP11-Cy5.5 and (GEBP11)_2_-ACP-Cy5.5 had high affinity and specificity for tumor vascular epithelial cells.

### *In vivo* NIR fluorescent imaging in subcutaneous gastric cancer xenograft models and biodistribution assays

**Figure 2:**
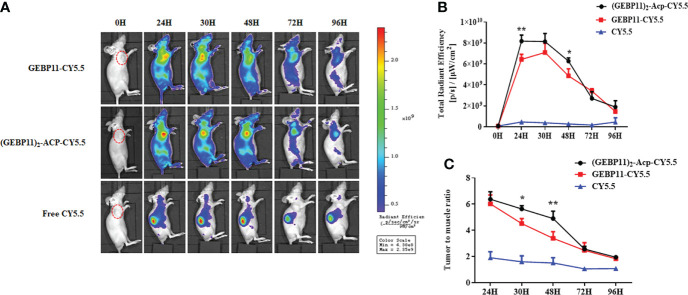
NIR fluorescent imaging of GEBP11 series probes in subcutaneous tumor models. **(A)** *In vivo* continuous observations (0-96 h) of SGC7901 bearing xenografts after intravenous administration of series probes by IVIS imaging system. (GEBP11)_2_-ACP-Cy5.5 probe exhibited more specific tumor targeting and longer retention, while the fluorescence signal mainly focused on kidney in the negative control (Free Cy5.5) group. **(B)** Dynamic changes of total fluorescence signal in the tumor site. **(C)** Comparison of tumor-to-muscle ratio of different probes. All data were expressed as mean ± SD. *p < 0.05; **p < 0.01.

The subcutaneous gastric cancer xenograft mice were used to test efficacy of the GEBP11 probes for live fluorescence imaging. NIR fluorescence signal could be detected at the tumor sites after injection with GEBP11-Cy5.5 or (GEBP11)_2_-ACP-Cy5.5. Free Cy5.5 accumulated mainly in the kidneys, and no significant signal was detected at the tumor sites (**Figure 2A**). At the tumor sites, the NIR fluorescence signal of (GEBP11)_2_-ACP-Cy5.5 peaked at 24h, gradually declined at 30h and 48h, then dropped markedly at 96h (**Figure 2B**). GEBP11-Cy5.5 fluorescence signal increased rapidly at 24h, peaked at 30h and decreased sharply at 48h (**Figure 2B**). To assess the relative signal intensity, tumor to muscle ratio (TMR) of the signal intensity was calculated. Both probes had the highest TMR value at 24h (**Figure 2C**). Overall, (GEBP11)_2_-ACP-Cy5.5 showed stronger and prolonged fluorescence signal than GEBP11-Cy5.5, suggesting that (GEBP11)_2_-ACP-Cy5.5 was more efficient and durable for live imaging (**Figure 2C**).

**Figure 3:**
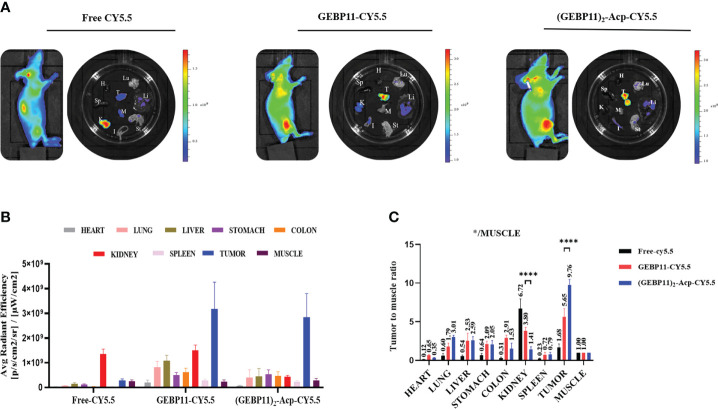
Biodistribution study of free Cy5.5, GEBP11-Cy5.5 and (GEBP11)_2_-Acp-Cy5.5 group *in vivo*. **(A)** Representative fluorescence images of the subcutaneous tumor-bearing mice and excised organs at 24 h post-injection, Color bar Units: [p/s/cm^2^/sr]/[μW/cm^2^]. **(B)** Average signal intensity of main organs in each group of mice. **(C)** Quantification of SBR profiles (tumor to muscle) of nude mice administered GEBP11 series probes at 24h. ****P < 0.001, n=3. *Abbreviation label of main organs: H, Heart, Lu, Lung, K, Kidney, Mu, Muscle; Sp, Spleen; Li, Liver; In, Intesinal; G, Gastric; T, Tumor.

To further validate the distribution of the NIR probes *in vivo*, tumors xenografts and major organs were harvested at 24h after the injection and underwent ex vivo fluorescence imaging. As shown in **Figure 3**, majority of free Cy5.5 was found in the kidney, without enrichment in the tumor. GEBP11-Cy5.5 was enriched in the tumor, with kidney showing moderate enrichment. Significant enrichment of the (GEBP11)_2_-ACP-Cy5.5 was found in the tumor, comparing with other normal organs. Tumor-to-muscle ratios of (GEBP11)_2_-ACP-Cy5.5 was 9.76, significantly higher than GEBP11-Cy5.5 (5.65). All these findings suggested that (GEBP11)_2_-ACP-Cy5.5 had higher specificity and better tumor-to-background contrast for *in vivo* imaging.

### *In vivo* NIR fluorescence imaging in orthotopic gastric cancer xenograft models

**Figure 4:**
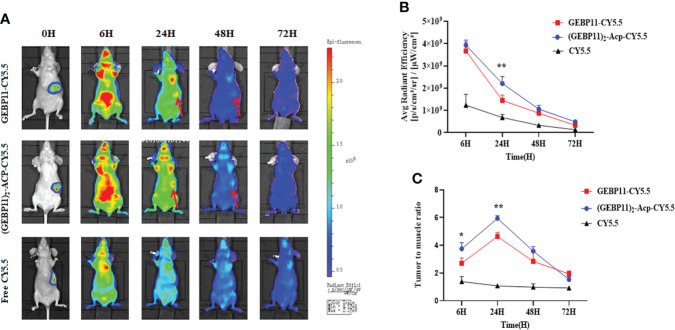
Targeting specificity of free Cy5.5, GEBP11-Cy5.5 and (GEBP11)_2_-Acp-Cy5.5. **(A)** fluorescence imaging of orthotopic tumor-bearing nude mice after injection with probes. **(B)** Total fluorescence signal emitted from the tumor site over time. **(C)** tumor-to-muscle ratio of the series probes over time. *p < 0.05; **p < 0.01.

To simulate the “natural” scenario, the GEBP11 probes were further evaluated for *in vivo* NIR fluorescence imaging in orthotopic gastric cancer xenograft mice. BLI was used to locate the tumor xenografts. As shown in **Figures 4A, B** stronger NIR fluorescence signal was detected at the tumor sites in mice injected with (GEBP11)_2_-ACP-Cy5.5 at 6h comparing with mice injected with GEBP11-Cy5.5 or free Cy5.5. Signal intensity for both (GEBP11)_2_-ACP-Cy5.5 and GEBP11-Cy5.5 probes peaked at 24h and then declined significantly at 48h, while signal intensity for free Cy5.5 dropped rapidly after 6h. Calculated tumor to muscle ratios (TMR) of the NIR signal intensity showed similar patterns (**Figure 4C**). The NIR fluorescence intensity curve of the (GEBP11)_2_-ACP-Cy5.5 NIR probe was smoother than GEBP11-Cy5.5, suggesting that binding of the (GEBP11)_2_-ACP-Cy5.5 was more rapid and stable from 6h to 24h after injection.

### Intraoperative fluorescence imaging guidance for gastric cancer resection

**Figure 5:**
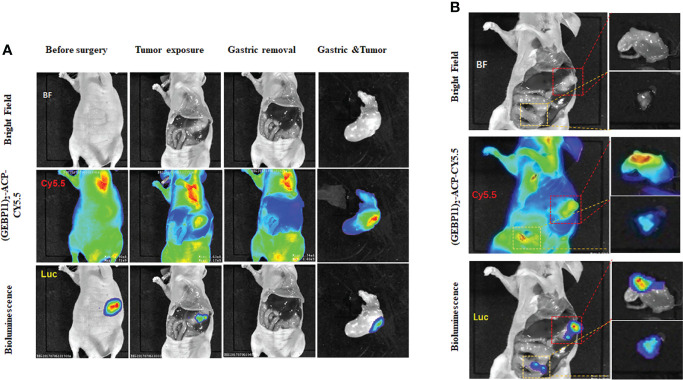
Intraoperative detection and resection of gastric cancer by (GEBP11)_2_-Acp-Cy5.5 probe. **(A)** Representative bioluminescence imaging (BLI) and fluorescence images of mice before and after surgery in orthotopic xenografts models under fluorescence guidance; **(B)** Intraoperative detection of peritoneal metastasis detected by white light imaging, BLI and molecular fluorescence-guided surgery (The red box represents gastric cancer in situ, the white box represents peritoneal metastasis location).

To assess the potential utility of (GEBP11)_2_-ACP-Cy5.5 to aid tumor resection, real-time intraoperative fluorescence was used to guide surgical resection of gastric cancer in orthotopic gastric cancer xenograft model. Under white light, xenograft tumors seemed to be deeply invasive and the margins between tumor and surrounding normal tissues were hard to identify. Under NIR fluorescence imaging, the tumor margins were located, and tumors were removed (**Figure 5A**). Interestingly, (GEBP11)_2_-ACP-Cy5.5 also detected peritoneal metastatic lesions indistinguishable under white light (**Figure 5B**). Metastatic lesions were also removed. After resection, the surgical beds and resected specimens were examined by NIR fluorescence imaging to ensure complete resection. There was a significant consistence between NIR findings and BLI imaging. No residual cancer lesion could be detected by BLI after NIR-guided resection.

**Figure 6:**
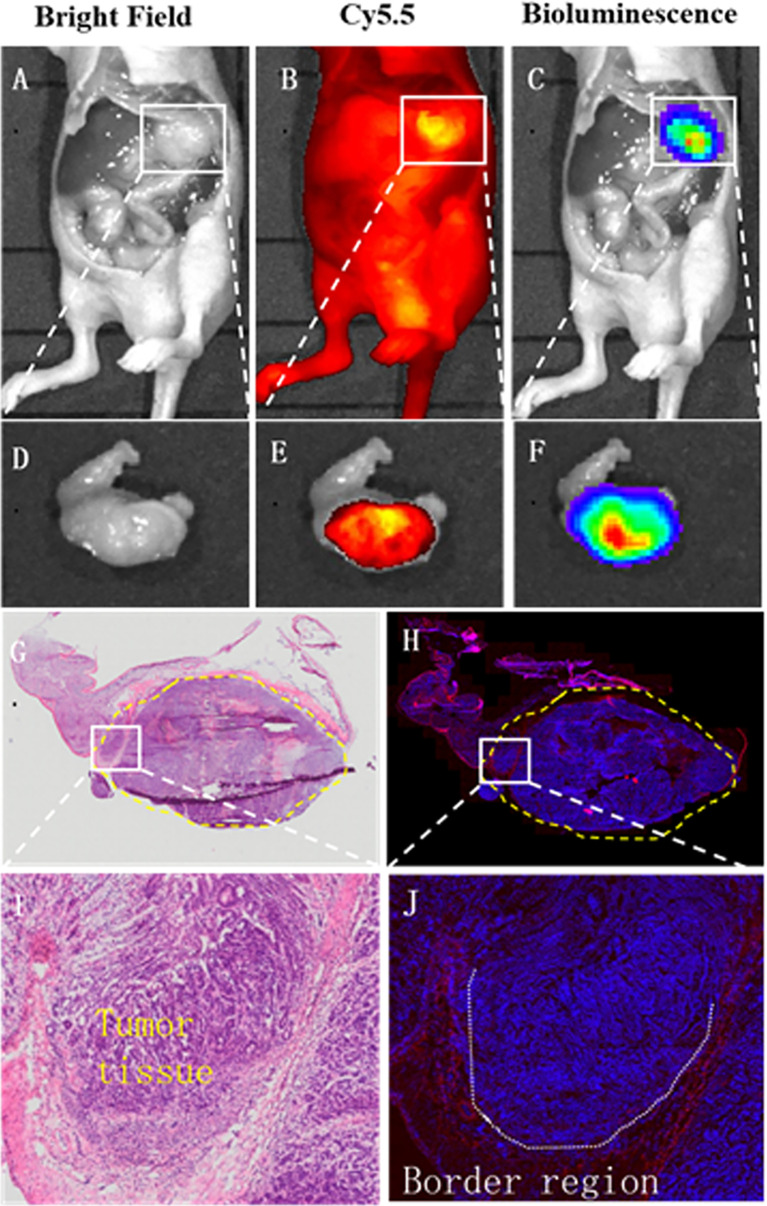
Intraoperative tumor margin assessment by (GEBP11)_2_-Acp-Cy5.5 probe. Compared with bright light imaging **(A, D),** (GEBP11)_2_-Acp-Cy5.5 probe enhanced tumor to normal tissue contrast under fluorescence imaging *in vivo* and *vitro* **(B, E),** which was confirmed by bioluminescence imaging **(C, F)**. **(G, H)** Immunohistochemical staining and fluorescence imaging showed a clear tumor positive circumferential resection margin (yellow dotted line). **(I)** Further microscopic histological analysis of the resected tissue clearly visualized border regions between tumor and surrounding healthy tissues, which was consistent with fluorescence signals of (GEBP11)_2_-ACP-Cy5.5 **(J)**.

**Figure 7:**
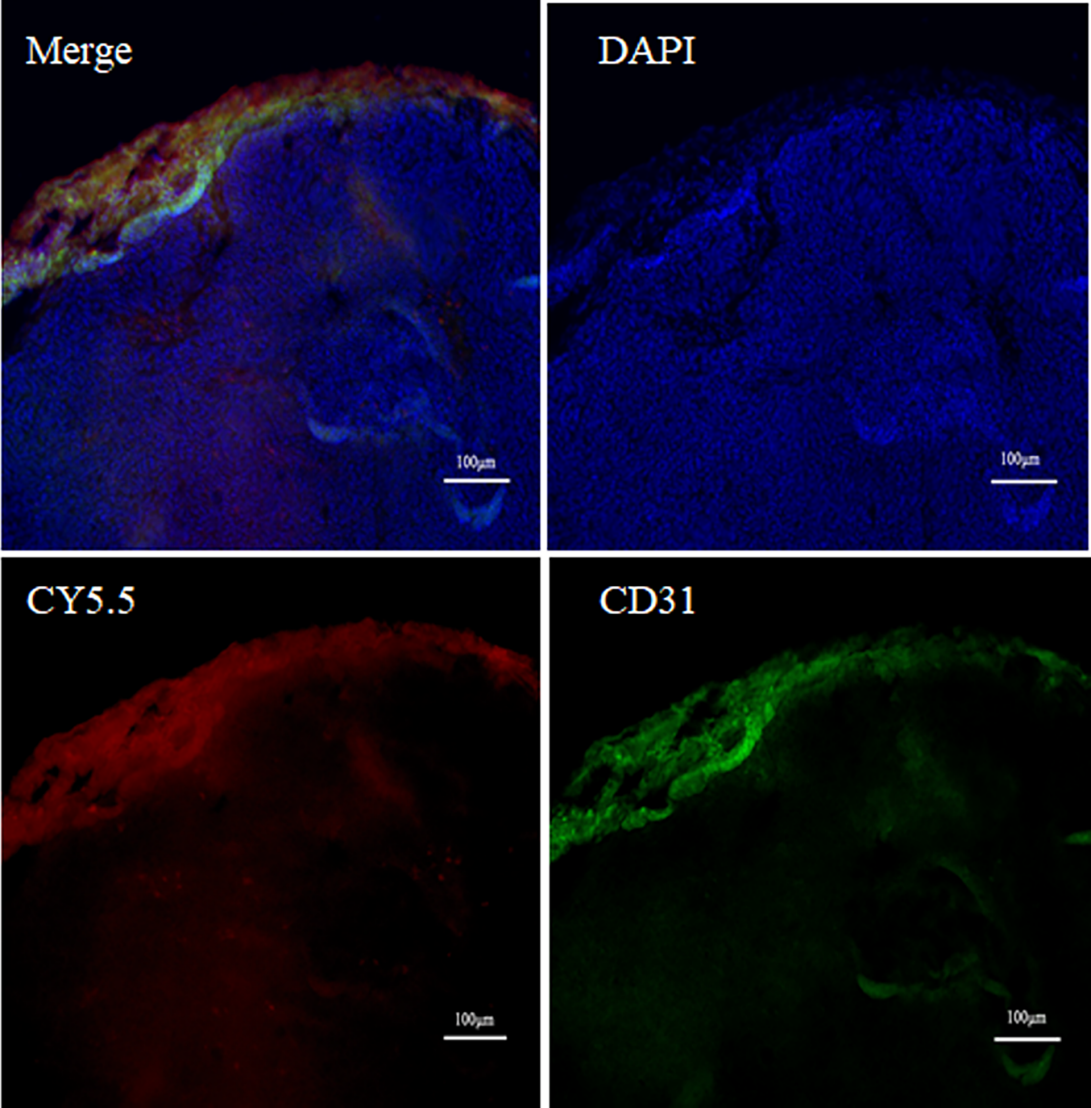
Vascular targeted co-location fluorescence probe imaging. Double labeling fluorescence using CD31 (green) and (GEBP11)_2_-Acp-Cy5.5 (red) probe showed tumor vessels in the peripheral area near the tumor edge.

After surgery, the margins between tumor and normal tissue were determined by NIR and BLI, and then histological examination was used to confirm the margins. Macroscopically, the margin defined by NIR fluorescence on macroscopic specimen correlated well with the H&E staining; Microscopic examination also confirmed the demarcation by NIR probe (**Figure 6**). By confocal fluorescence microscopic examination, fluorescence signals of (GEBP11)_2_-ACP-Cy5.5 was found to co-localize with CD31 on the tumor vascular epithelial cells that delineated the tumor margins (**Figure 7**). Taken together, all these findings suggested that NIR-dye-labeled (GEBP11)_2_-ACP could serve as an optical imaging probe for real-time intraoperative NIR guidance in gastric cancer resection.

## Discussion

Molecular imaging is playing increasingly important roles in cancer diagnosis and treatment, especially in cancer surgery. Compared with traditional imaging techniques, intraoperative fluorescence imaging shows new possibilities and promising potential in cancer care. Currently, diverse biological imaging agents such as radionuclide, iron nanoparticles, and NIR agents have been designed for tumor imaging and/or therapy of gastric cancer (24, 25). A nanocolloid radiopharmaceutical reagent has been tested for intraoperative visualization of SLN in gastric cancer patients (26). The radionuclide detected SLN with high sensitivity and specificity but could not distinguish metastatic from nonmetastatic lymph nodes. The need for radiometry also complicated the procedure. Moreover, it is hard for the nanoparticles to permeate and transfer to the tumor sites due to increased interstitial fluid pressure in gastric cancer. Several NIR imaging reagents have also been reported. Among them, ICG fluorescence imaging (ICG-FI) is a well-established modality in the SLN mapping and image-guided surgery of gastric cancer (27). Nevertheless, ICG-FI suffers from several intrinsic drawbacks such as its non-specificity, instability and limited quantum yield (27, 28). Specific tumor antigens such as HER2 and carcinoembryonic antigen (CEA) have been targeted for live imaging during gastric cancer surgery (29–32). However, only a small portion of gastric cancer express HER2 and CEA, which limited their use.

Tumor stromal cells serve as potential better targets for fluorescence guided oncologic surgery. Aggressive cancers usually have high stromal content, which often primarily locate at the periphery/invasive front of the tumor. Unlike tumor antigens, tumor stromal markers do not rely on specific genetic variation and seem to be more “universal”. Neo-angiogenic endothelial cells is the first tumor stromal cell to be exploited for imaging (33). Antibodies, peptides, and small molecules targeting tumor vascular endothelial cells have been developed. IRDye800CW labeled with bevacizumab has been used in various fluorescence molecular imaging trial targeting tumors and peritoneal carcinomatosis that overexpress vascular endothelial growth factor (12, 34, 35). Antibodies have large mass and complexed structure, making them unsuitable for the targeting of solid tumors. The properties of peptide, such as small size, high specificity, and easier tissue clearance make them more preferrable for tumor imaging. The stability and half-life of the targeting peptides could be further improved by multivalent modification while preserving the correct conformations (36, 37).

In our study, we used a peptide specific for gastric cancer vasculature for tumor imaging. Both mono and dimeric GEBP11 polypeptides showed good affinity and specificity for gastric cancer vascular endothelial cells. Previous studies indicated that multivalent modification could increase circulation time and lower renal clearance of the probes (19, 38). In the present study, dimeric GEBP11 peptide showed better accumulation in tumors, prolonged signals and reduced renal accumulation *in vivo*. Therefore, (GEBP11)_2_-ACP-Cy5.5 was further tested for intraoperative fluorescence imaging. Under NIR, tumor margins and peritoneal metastases could be clearly visualized during surgery. Complete resection was achieved in all animals. By examining the surgical specimens, NIR signals correspond well with the histological findings, which clearly delineated tumor margins and metastatic lesions. The specificity of the probes for tumor vascular endothelial cells was confirmed by CD31 co-staining. Taken together, we developed a specific NIR probe for gastric cancer imaging, which showed promising potential for fluorescence guided oncologic surgery.

Although the findings of this study are encouraging, there are some limitations. First, GEBP11 peptide receptor on vascular endothelial cell is still unclear, but several candidate receptor molecules have been acquired. Further work needs to be conducted to understand the interaction between GEBP11 peptide and its ligand. Second, we used Cy5.5 to label the probes. Currently the use of cyanine dyes is still mostly restricted in the laboratory. IRDye800CW has low auto-fluorescence, high spatial resolution and has been used to label drugs and probes in clinical trials (39). To facilitate clinical translation, labeling (GEBP11)_2_-ACP with IRDye800CW for intraoperative imaging should be investigated. Third, though specificity and binding affinity of GEBP11 for human gastric cancer vasculature have been confirmed *in vitro* (20). *In vivo* distribution of the probes in human should be invested before clinical application. Besides, the usefulness of the probes for metastatic gastric cancer should also be further studied. Other models of gastric cancer with distal metastasis to lung, liver or other organs should be used.

## Conclusion

In summary, the present study demonstrates the potential of a specific targeting probe, (GEBP11)_2_-ACP-Cy5.5, for highlighting tumor lesions with high contrast during fluorescence-guided surgery, which may be used for the real-time image-guided oncologic surgery of gastric cancer.

## Data availability statement

The original contributions presented in the study are included in the article/**Supplementary Material**. Further inquiries can be directed to the corresponding authors.

## Ethics statement

The animal study was reviewed and approved by Institute of Animal Use Committee of Air Force Medical University. Written informed consent was obtained from the owners for the participation of their animals in this study.

## Author contributions

All of authors have contributed the work. Contributing to conception and design: JZ, CG, XinZ, ZT. Carrying out experiments: JZ, ZT, MT, XiaZ. Analysis and interpretation of the data: JZ, ZT, CG. Drafting the manuscript: JZ, ZT, CG. Critical revision of the article for important intellectual content: JZ, CG, SL. Final approval of the article: XinZ, JZ, CG. Scientific advisors: JZ, CG, XinZ. Financial support: JZ, SL. They have seen and approvaled the content of the manuscript submitted for publication.

## Funding

This work was supported by the National Natural Science Foundation of China (No. 81402467, No. 81472778, No.81627807 and No. 82000506).

## Conflict of interest

The authors declare that the research was conducted in the absence of any commercial or financial relationships that could be construed as a potential conflict of interest.

## Publisher’s note

All claims expressed in this article are solely those of the authors and do not necessarily represent those of their affiliated organizations, or those of the publisher, the editors and the reviewers. Any product that may be evaluated in this article, or claim that may be made by its manufacturer, is not guaranteed or endorsed by the publisher.

## Supplementary material

Supplementary Figure 1 Chemical structure and MS identification of Cy 5.5 labeled GEBP11 probes.

Supplementary Figure 2 Stability of GEBP11-Cy5.5 and (GEBP11)_2_-Acp-Cy5.5. *In vivo* binding affinity and targeting properties of probes with different storage duration (**A, D:** 7day, **B, E:** 2month, **C, F:** 6month) were tested in subcutaneous tumor models. Fluorescence images of mice and excised organs were collected 24h after tail vein injection (H, Heart, Lu, Lung, K, Kidney, Mu, Muscle; Sp, Spleen; Li, Liver; In, Intestinal; G, Gastric; T, Tumor).

Supplementary Figure 3 NIR fluorescent imaging of GEBP11 probes injection within 24 hours in subcutaneous tumor models. (A) *In vivo* continuous observations (24 h) of SGC7901 bearing xenografts after intravenous administration of series probes by IVIS imaging system. (B) Quantification of average fluorescent efficiency changes with time dependent in the tumor site. (C) Comparison of tumor-to-muscle profiles between (GEBP11)_2_-ACP-Cy5.5 & GEBP11-Cy5.5 probe. ****p < 0.001.
