# Supplementary figures and images for: Near-infrared-dye labeled tumor vascular-targeted dimer GEBP11 peptide for image-guided surgery in gastric cancer
Source: Front Oncol. 2022 Nov 24;12:885036. doi: 10.3389/fonc.2022.885036 (PMC9730820; doi:10.3389/fonc.2022.885036)

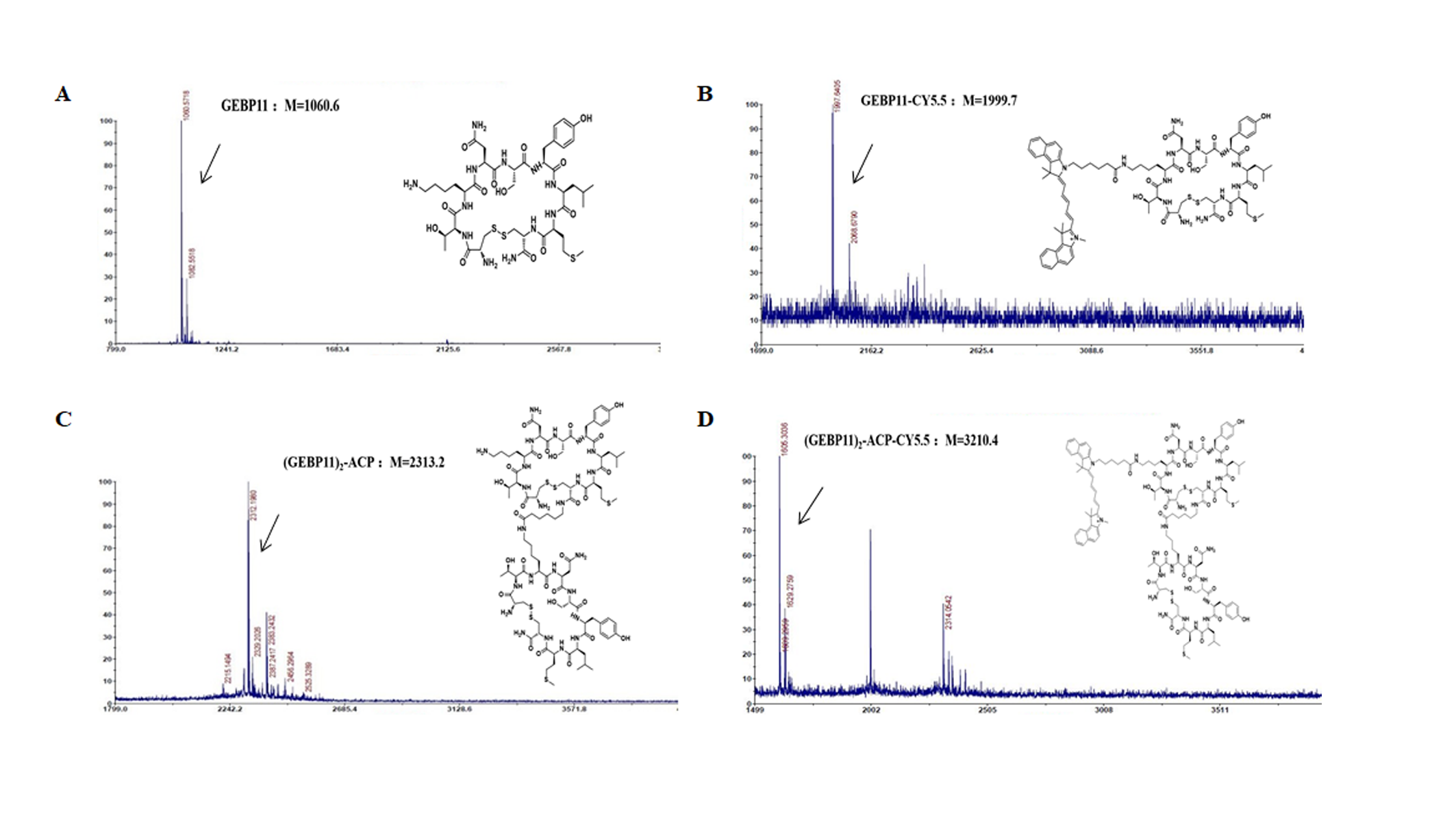

Supplement: Supplementary Figure 1 — Chemical structure and MS identification of Cy 5.5 labeled GEBP11 probes. [file Image_1.tif]

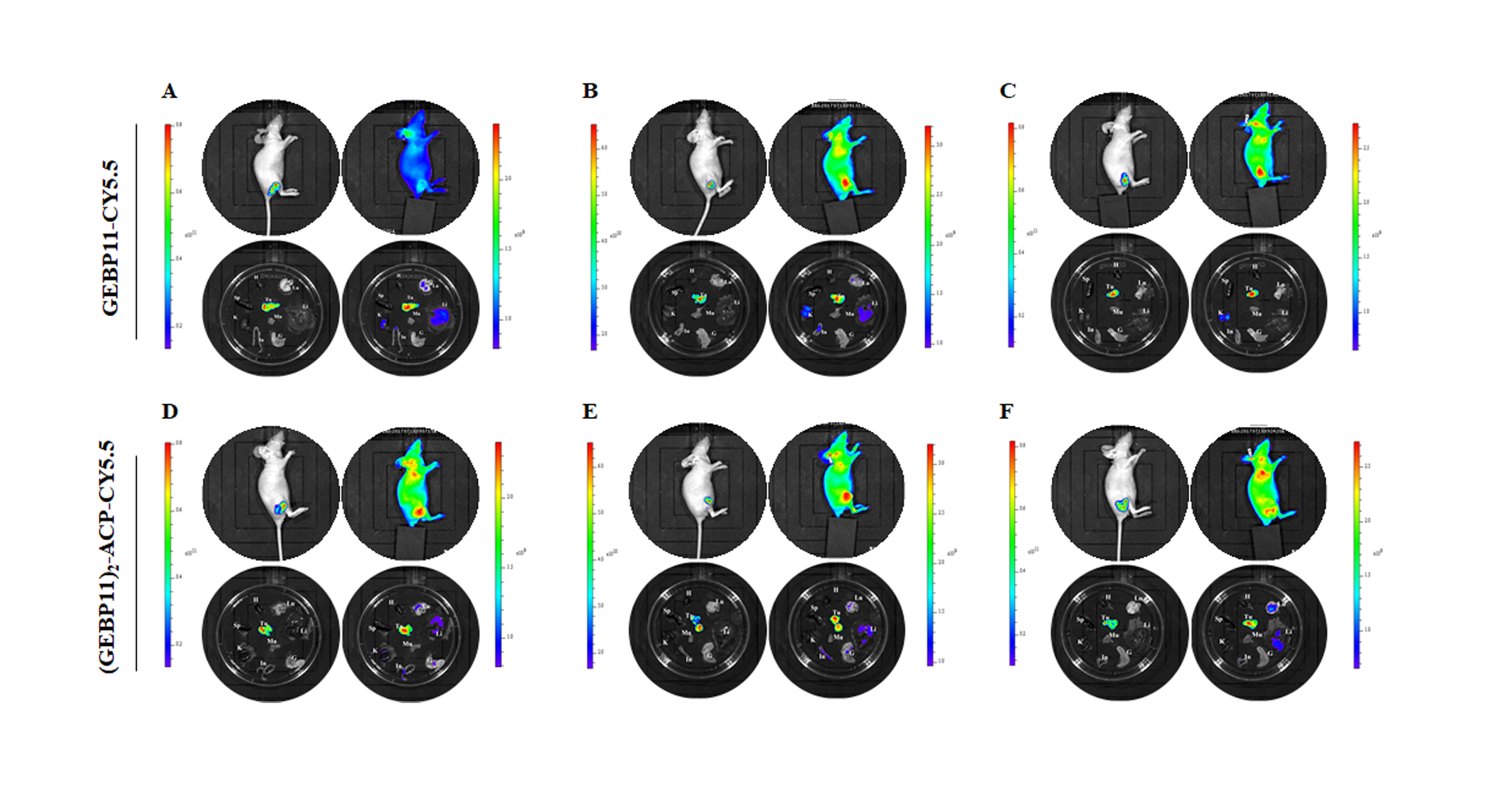

Supplement: Supplementary Figure 2 — Stability of GEBP11-Cy5.5 and (GEBP11)2-Acp-Cy5.5. In vivo binding affinity and targeting properties of probes with different storage duration (A, D: 7day, B, E: 2month, C, F: 6month) were tested in subcutaneous tumor models. Fluorescence images of mice and excised organs were collected 24h after tail vein injection (H, Heart, Lu, Lung, K, Kidney, Mu, Muscle; Sp, Spleen; Li, Liver; In, Intestinal; G, Gastric; T, Tumor). [file Image_2.tif]

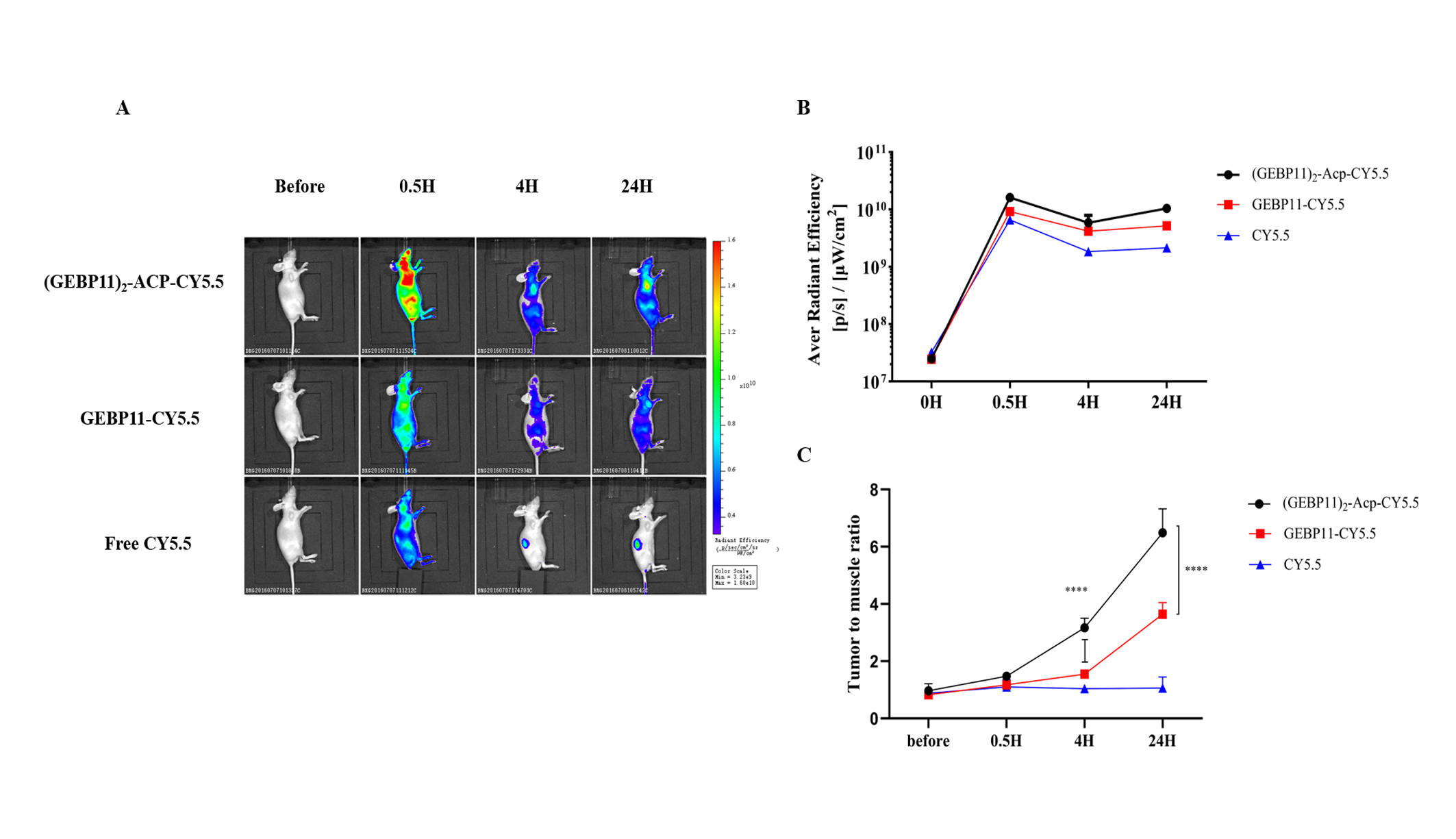

Supplement: Supplementary Figure 3 — NIR fluorescent imaging of GEBP11 probes injection within 24 hours in subcutaneous tumor models. (A) In vivo continuous observations (24 h) of SGC7901 bearing xenografts after intravenous administration of series probes by IVIS imaging system. (B) Quantification of average fluorescent efficiency changes with time dependent in the tumor site. (C) Comparison of tumor-to-muscle profiles between (GEBP11)2-ACP-Cy5.5 & GEBP11-Cy5.5 probe. ****p < 0.001. [file Image_3.tif]
